# LiDAR-Based Bridge Displacement Estimation Using 3D Spatial Optimization

**DOI:** 10.3390/s20247117

**Published:** 2020-12-11

**Authors:** Gichun Cha, Sung-Han Sim, Seunghee Park, Taekeun Oh

**Affiliations:** 1 School of Civil, Architectural Engineering and Landscape Architecture, Sungkyunkwan University, Suwon 16419, Korea; ckckicun@skku.edu (G.C.); ssim@skku.edu (S.-H.S.); 2 Department of Safety Engineering, Incheon National University, Incheon 22012, Korea

**Keywords:** light detection and ranging (LiDAR), terrestrial laser scanning (TLS), deflection, octree space partitioning (OSP), structural health monitoring (SHM)

## Abstract

As civil engineering structures become larger, non-contact inspection technology is required to measure the overall shape and size of structures and evaluate safety. Structures are easily exposed to the external environment and may not be able to perform their original functions depending on the continuous load for a long time. Therefore, in this study, we propose a method for estimating the vertical displacement of structures using light detection and ranging, which enables non-contact measurement. The point cloud acquired through laser scanning was rearranged into a three-dimensional space, and internal nodes were created by continuously dividing the space. The generated node has its own location information, and the vertical displacement value was calculated by searching for the node where the deformation occurred. The performance of the proposed displacement estimation technique was verified through static loading experiments, and the octree space partitioning method is expected to be applied and utilized in structural health monitoring.

## 1. Introduction

Bridges are key infrastructural components of transportation facilities, including roads and railways. Bridges are subjected to constant long-term loads owing to the daily passage of several vehicles. Sustained long-term loads degrade the bridge’s function and can lead to major damage if the bridge becomes deformed [1,2,3]. In addition, aging bridges become quite vulnerable to natural disasters, such as earthquakes and floods, due to deterioration in performance [4,5]. Therefore, bridges are essential structures that require regular inspections. Most bridges undergo regular inspections that incorporate contact-type sensors. Depending on the size of the bridge and the number of sensors installed, the amount of data collected can increase or decrease. Furthermore, if access to the bridge is difficult, the sensor cannot be installed. The limits of contact-type sensors make it very difficult to assess the safety of bridges. To address these problems, non-contact inspection techniques have been developed [6,7,8].

Non-contact measurement methods, such as image capturing using cameras, thermal imaging cameras, and laser scanning, are now being used for structural safety inspection. In the case of a camera, a separate target point must be installed to estimate the bridge displacement. In addition, multiple cameras must be installed, and the position of the fixed camera must not be changed. For example, if the camera position is changed owing to wind, a large error can occur in the bridge displacement estimation [9,10,11]. In the case of laser scanning, a target point and separate data network are not required for data acquisition. The necessary equipment for this procedure is a laser scanning device and a laptop computer, which allows the researcher to obtain data on the entire surface of the structure [12,13,14,15,16,17,18,19,20,21,22].

Light detection and ranging (LiDAR) systems, including laser scanning, are remote measurement systems that use lasers. LiDAR systems can acquire the overall shape of a structure with precise three-dimensional coordinates within a few minutes. Therefore, LiDAR systems can easily acquire the shape information of structures that are difficult to access. In this study, we propose a method for measuring the deflection of structures that can check and manage changes in the overall shape of structures in 3D space using LiDAR data.

## 2. Methods

This study uses LiDAR to acquire the shape information of structures from a long distance for long-term maintenance of structures. The main feature of LiDAR is fast acquisition of the entire shape of a structure. It does not require a separate wired network for data acquisition. LiDAR can acquire structural data at distances of approximately 300 m; hence, it is possible to acquire data on the entire surface, even for structures that are difficult to access. Therefore, LiDAR has frequently been used in structural displacement measurement studies.

There are three ways to obtain displacement estimation of bridge structures using laser scanning:

- grid-based displacement estimation,
- triangle grid-based displacement estimation, and
- displacement estimation based on the least-squares method.

First, grid-based displacement estimation creates a grid on a point cloud, and each coordinate point is replaced with a grid center point to estimate displacement. If the distribution of points is not uniform, this method generates an irregular shape and includes many errors. Second, triangle grid-based displacement estimation calculates displacement using three points on the same line. In this method, when a point is missing, the area of the triangle increases, and an error that is equal to the wrong area is included. Third, the least-squares method calculates the distance to each point by defining an arbitrary line. This method significantly reduces accuracy when there are missing points or when the point distribution is irregular.

In this study, the point cloud is used as a node in the proposed 3D space. An internal node is created through the octree spatial division algorithm, and the displacement of the structure is measured using the generated node.

### 2.1. Grid-Based Displacement Estimation

The grid mean approximation method is an extension of the concept of arithmetic mean to the spatial domain, and it has been applied experimentally to generate reference data for 3D data on a surface. This method includes grid point errors and forms grids on the x and y planes based on irregularly distributed spatial data. Thereafter, the z coordinates of the data in the grid are averaged to determine the representative point of each grid [13,23].

The representative point is set as reference data in the center of the grid, as shown in Equation (1), where *Z_ji_* represents the *Z* coordinate value of the *i*-th coordinate data included in the *j*-th space.

(1) $$Z_{j}=\;\frac{1}{n_{j}}\;\overset{n_{j}}{\underset{i=1}{\sum }}Z_{ji}\;\;\;\;\;\;\;\;\;\left(i=1\;\mathrm{to}\;n_{j},\;j=1\;\mathrm{to}\;m\right)$$

### 2.2. Triangle Grid-Based Displacement Estimation

The triangular grid approximation is a method that performs the linear trigonometric interpolation method in reverse. This method uses a multiple regression analysis technique and calculates the displacement of a structure using structural information such as strain, stress, displacement, and z-coordinates [24].

This technique uses the strain and shape functions of three vertices, 1, 2, and 3, of the triangle on the plane. The strain for a point inside the triangle can be expressed by Equation (2). The shape functions *N*_1_, *N*_2_, and *N*_3_ are calculated through natural coordinates, and the point p defined on the *xy* plane can be expressed as Equation (3).

(2) $$\varepsilon =\;f\left(N_{1},\;N_{2},\;N_{3}\right)=N_{1}\varepsilon_{1}+\;N_{2}\varepsilon_{2}+\;N_{3}\varepsilon_{3}$$

(3) $$P\left(x,y\right)=p\left(\varepsilon ,\;Z\right)$$

### 2.3. Displacement Estimation Based on the Least-Squares Method

The least-squares plane technique determines a function that can define a correlation between data measured through experiments. Within the multiple linear regression analysis, the deviation of the coordinate values included in the plane from the plane is minimized by applying the least-squares theory to form a reference plane [8,25].

The approximation process determines the regression coefficient of the function that minimizes the square of the difference between the multi-linear regression function values reflecting the characteristics of the data and measured data values. When the function is determined, the vertical displacement value of the Z axis is calculated by comparing it with the measured data value. Z is estimated by the explanatory coefficients, $N_{1},\;N_{2},\;N_{3}\ldots N_{m}$, and regression coefficients, $\beta_{1},\;\beta_{2},\;\beta_{3}\ldots \;\beta_{i}$, as shown in Equation (4).

(4) $$z=\;\beta_{0}+\;\beta_{1}N_{1}\ldots +\beta_{m}\beta_{m}+e$$

### 2.4. Displacement Estimation Based on Octree Space Partitioning Algorithm

The octree space partitioning (OSP) algorithm is used to accurately represent objects in the 3D space. The octree refers to the format of the data storage structure, and it connects the upper and lower spaces by subdividing the space [26].

**Figure 1:**
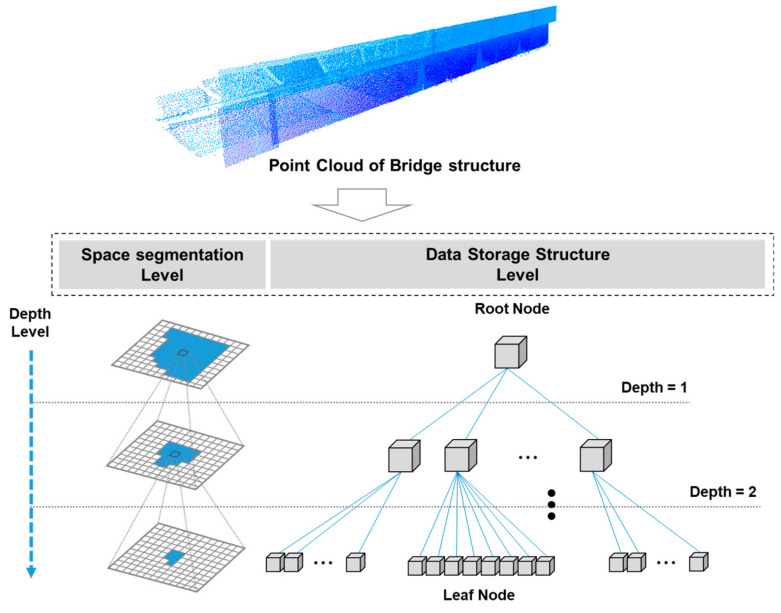
Storage structure of point cloud data based on octree space partitioning (OSP) algorithm.

The connected space exhibits a parent–child relationship, as shown in Figure 1. The uppermost space is called a root node, and the lowest space is called a leaf node. In this study, the vertical displacement was estimated by relocating the point cloud data in a 3D space and then dividing it in detail to search for the change in the position of the leaf nodes.

To use the octree data structure, a 3D object must first be created. An object is constructed using a voxel, the smallest unit of 3D spatial representation. Voxels are volumetric elements and represent normal grid unit values in 3D space. The word “voxel” combines the words “volume” and “pixel” and expands the concept of volume pixel into three-dimensional space. A voxel does not have coordinates in space but recognizes its position with respect to other voxels.

## 3. Estimation of Vertical Displacement Using 3D Spatial Optimization

**Figure 2:**
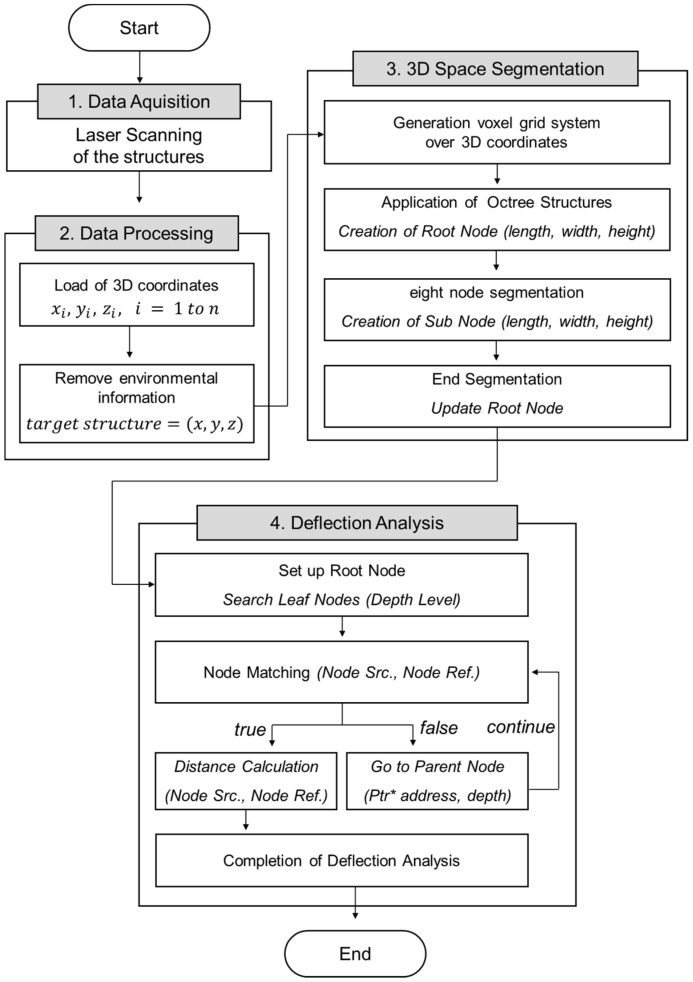
Vertical displacement estimation process using 3D spatial optimization.

In this section, an experiment was conducted to compare the three mathematical techniques and displacement estimation techniques using the 3D spatial optimization proposed in this study. Figure 2 shows the research process for estimation of vertical displacement. After removing the environmental information from the scan data, the 3D coordinates were rearranged with the voxel grid system. When data arrangement is complete, the space is continuously divided and nodes are connected. Each node has location information, and it becomes possible to move between connected nodes. Finally, the node where the change occurred was searched and calculated as the vertical displacement value.

### 3.1. Data Acquisition

The test specimen was a high-performance steel (SS400) section installed and used in an actual bridge structure. The laser scanning equipment was installed at a distance of 7 m from the SS400 specimen, and the load test was performed using a universal testing machine (UTM) to measure the displacement after applying a constant load to the specimen. In addition, a linear variable differential transformer (LVDT) sensor was installed under the specimen to obtain reference data for comparison with data from laser scanning. The load was applied in four stages: 0.8, 1.6, 2.4, and 3.2 kN. The laser scanning equipment was a Leica ScanStation C5 model; detailed performance information is summarized in Table 1.

**Figure 3:**
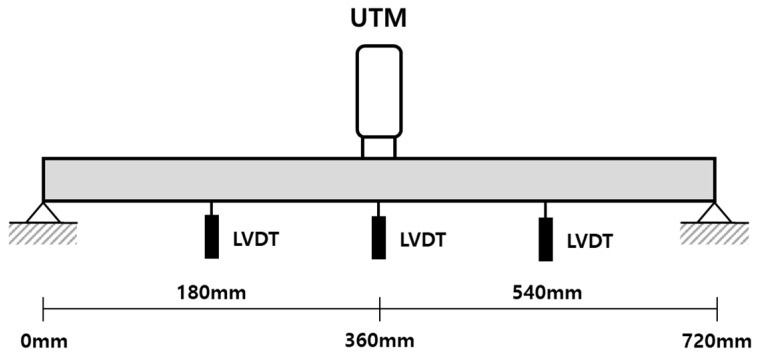
Linear variable differential transformer (LVDT) installation point.

The detailed sizes of the specimens are listed in Table 2. The three specimens were of 8, 10, and 12 mm each, and the length between both ends of the support was 720 mm. LVDTs were used, and vertical displacement was measured at the ¼, ½, and ¾ length points, as shown in Figure 3. The LVDT used in the experiment contained an error rate of ±0.03 mm.

**Figure 4:**
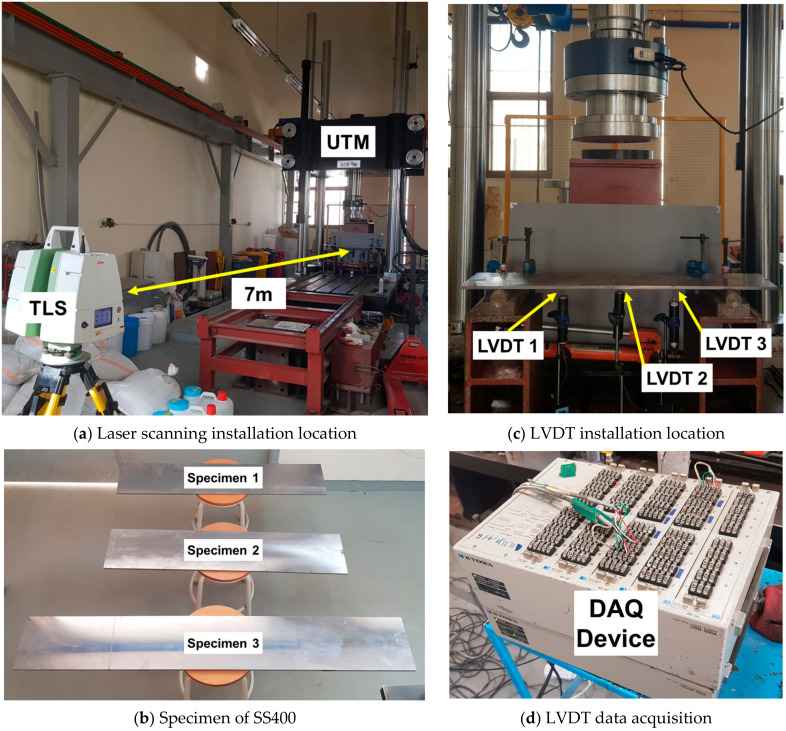
Laser scanning installation and experiment environment for static loading experiment.

The static loading experiment environment can be seen in Figure 4. Laser scanning was installed at a distance of 7 m. The theoretical value was derived based on the elastic deflection theory, as shown in Equation (5), and compared with the LVDT value. In the case of the two specimens (10 and 12 mm), the difference between the theoretical and measured values was 0.1 to 0.5 mm. In the case of a specimen (8 mm), the theoretical and measured values showed a difference of 1.0 to 2.0 mm, which can be confirmed in Table 3.

The reason for the different values is that the weight of the header of the UTM equipment is added to the set load, and it appears that a displacement larger than the theoretical value has occurred.

(5) $$Deflection_{max}=\;\frac{PL^{3}}{48EI}$$

Three LVDTs were fixed, and the vertical displacement values were measured by installing LVDTs at 18, 36, and 54 cm. The vertical displacement value was measured at three points, and the measured values are listed in Table 4. The LVDT installed at 36 cm measured the displacement at the position where the load was directly applied to the center point of the specimen.

### 3.2. Data Processing

Laser scanning captures the shape information of all objects within the measurement range. Therefore, accurate analysis results can be obtained by removing the information that is not within the range of the specimen. This was performed using the software provided by the scanner manufacturer; the relevant information range was selected, and the rest of the information was removed.

**Figure 5:**
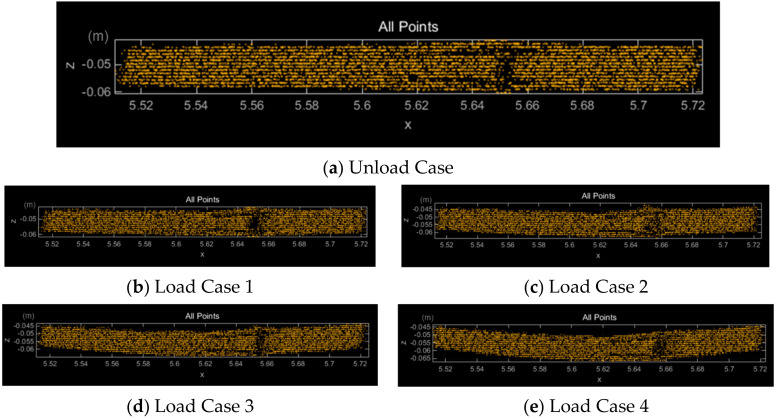
Scanning data of a specimen (10 mm) for each load.

The point cloud measured via laser scanning is shown in Figure 5. The data consisted of a set of numerous points. The vertical displacement value was measured by applying the terrestrial laser scanning (TLS) (Grid), TLS (Tri), and TLS (LSP) techniques. It was also measured using the TLS (OSP) technique proposed in this study.

### 3.3. D Space Segmentation

The measured laser scanning data generated internal nodes through the spatial division algorithm, as shown in Table 5. The generated octree model checks the node to find the deformed point; if it finds the deformed node, it calculates the position from the reference node to estimate the vertical displacement value. The internal address system is constructed in a structure that can be searched by connecting spatial information in the object model and creating numerous internal nodes that exhibit a parent-child relationship. The computer performance used for data analysis is summarized in Table 6.

The octree spatial division algorithm proceeded in two stages. The first step was to rearrange the point cloud in a three-dimensional space. In the second step, the vertical displacement was estimated by searching for node movement.

**Figure 6:**
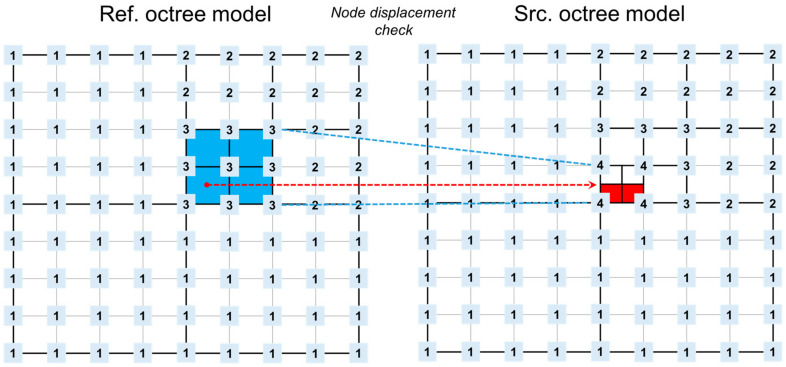
Detecting variant nodes viewed from the perspective above the root node.

In Figure 6, nodes that did not exist in the Ref octree model (blue area) exist in the Src octree model (red area). The red area is recognized as the location where the displacement value appears. The vertical displacement was estimated by searching for node movement between the reference model and the source model with deformation, as summarized in Table 5.

The models created through the 3D spatial division algorithm can be seen in Table 7 and Table 8. Nodes of the model without load are marked in blue, and areas where node movement occurred are marked in red. The node movement was evident in the 8 mm specimen, and the confirmed node changes are marked in red, even in the 10 and 12 mm specimens.

### 3.4. Deflection Analysis

Laser scanning was performed eight times for each load case, and scanning data of 40 scans per specimen were obtained, including those for the case without load. Four displacement estimation techniques were applied using the point cloud of the specimen. The maximum displacement occurred at the center of the specimen at 36 cm, and the results are summarized in Table 9.

**Figure 7:**
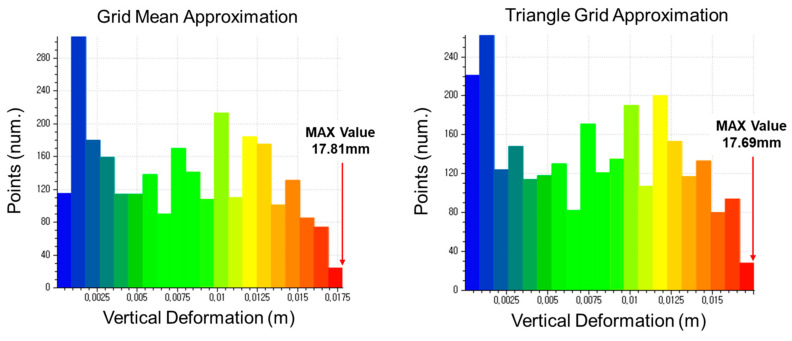

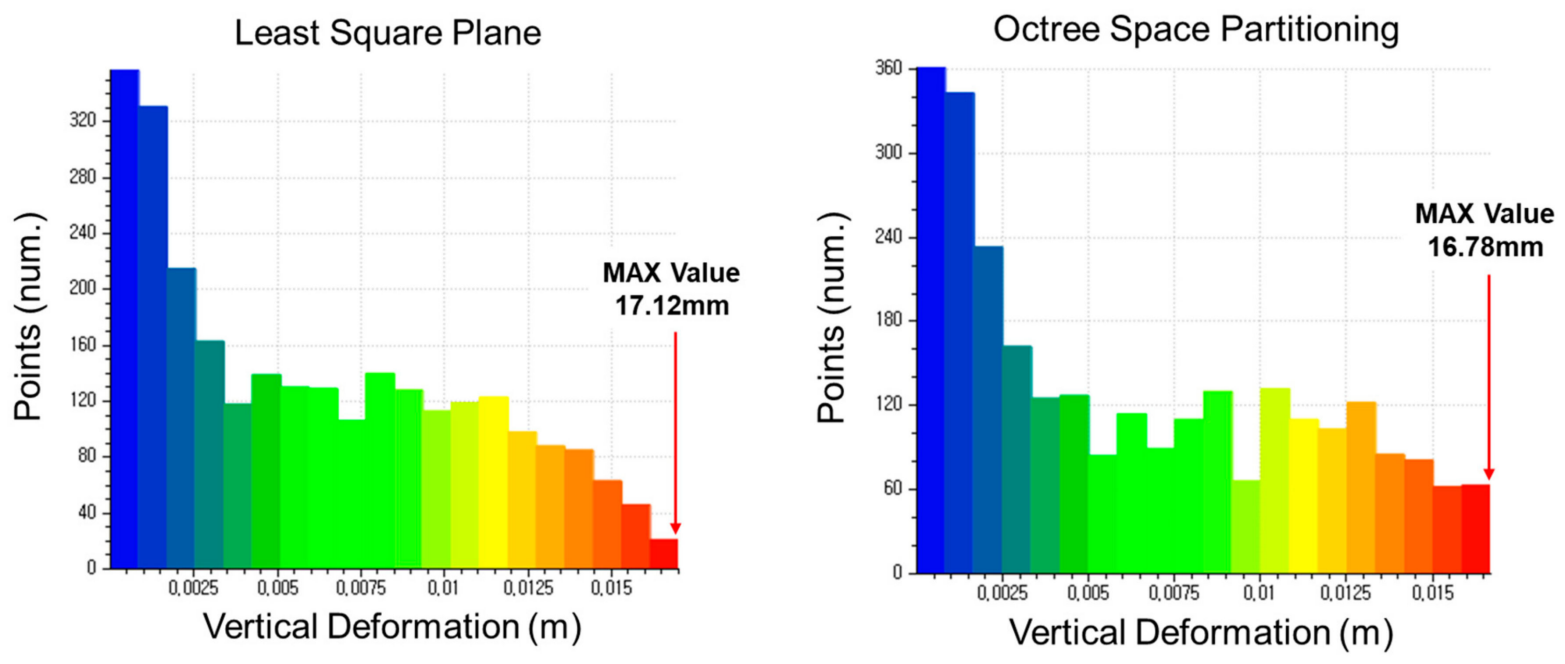
Classification of vertical displacement of the 8 mm specimen (LC4).

**Figure 8:**
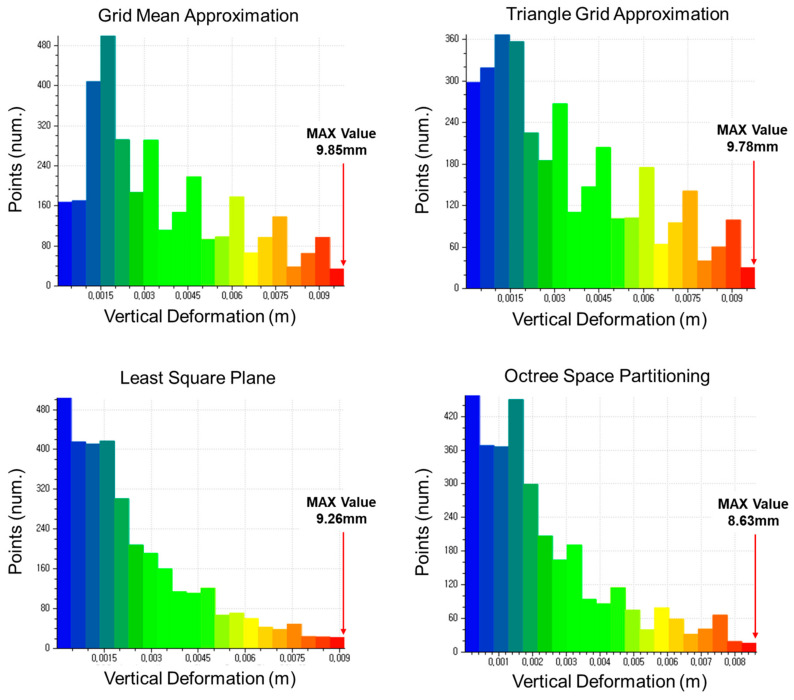
Classification of vertical displacement of the 10 mm specimen (LC4).

**Figure 9:**
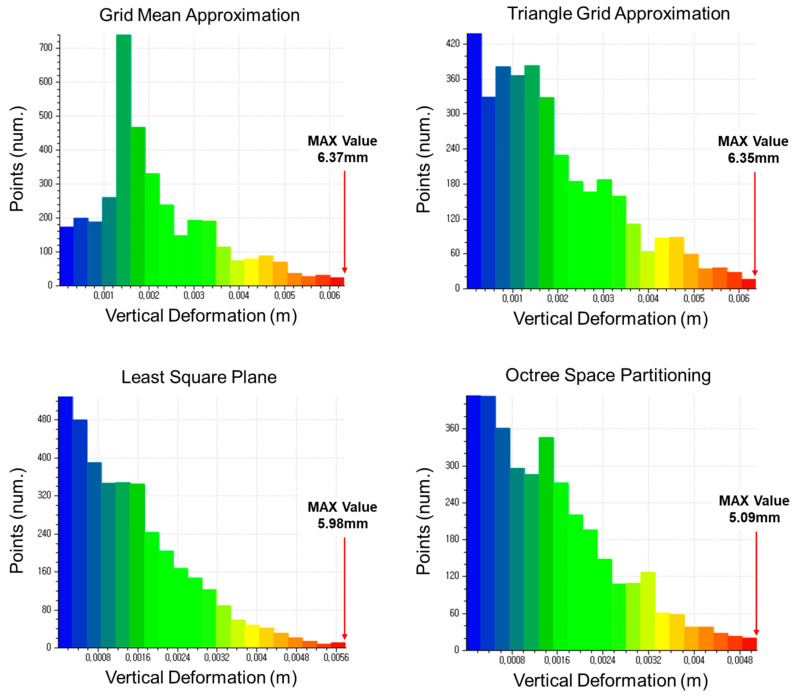
Classification of vertical displacement of the 12 mm specimen (LC4).

The displacement estimation results of each method were classified into 24 classes. The 24th class represents the maximum displacement value. The classes of the 8, 10, and 12 mm specimens can be seen in Figure 7, Figure 8 and Figure 9, respectively.

**Figure 10:**
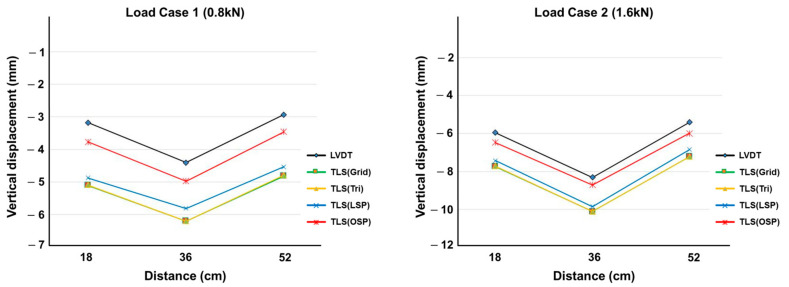

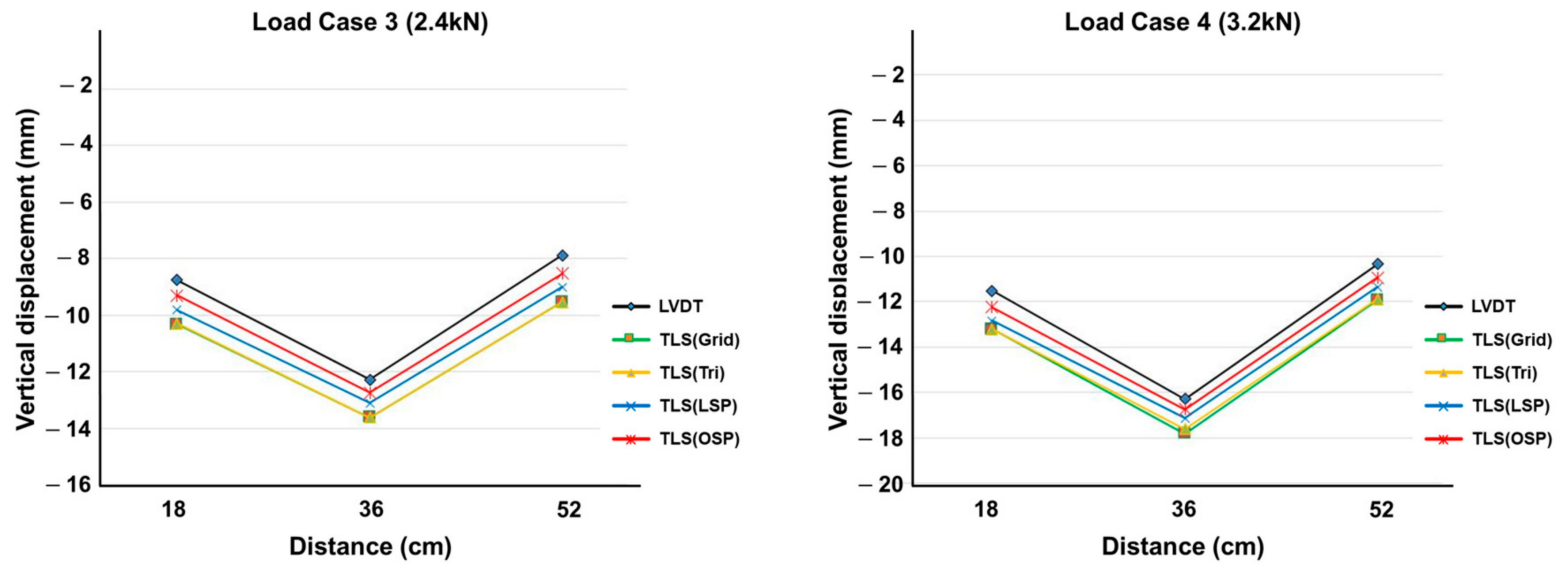
Comparison of vertical displacement between LVDT and the four methodologies (8 mm specimen).

**Figure 11:**
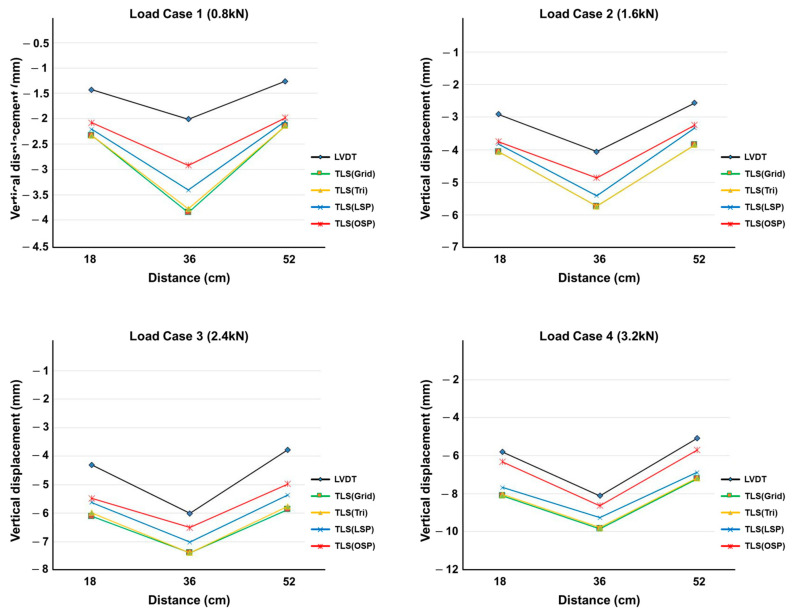
Comparison of vertical displacement between LVDT and the four methodologies (10 mm specimen).

**Figure 12:**
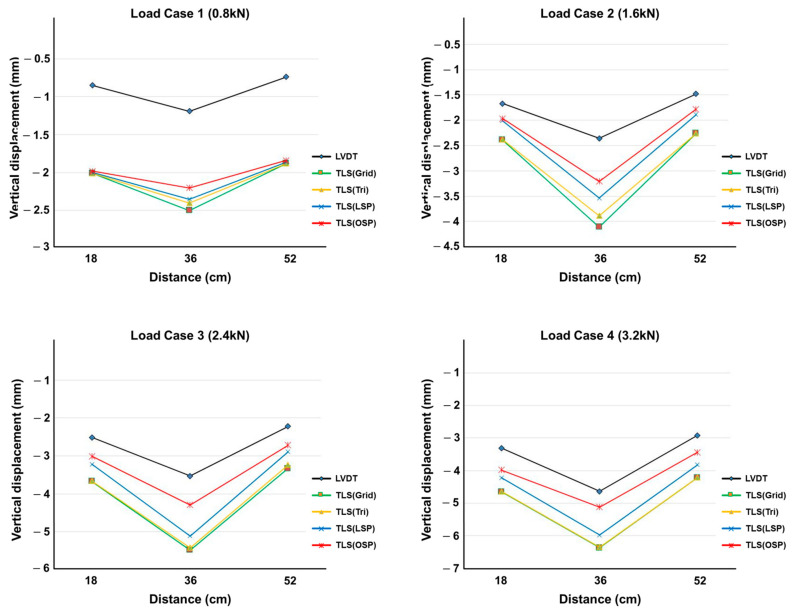
Comparison of vertical displacement between LVDT and the four methodologies (12 mm specimen).

The vertical displacement values estimated using the four methodologies were compared with the LVDT measurements. The TLS (Grid) and TLS (Tri) techniques showed similar results in all displacement sections, and TLS (LSP) showed larger displacement values than TLS (OSP) did. Figure 10, Figure 11 and Figure 12 show the same pattern: as the vertical displacement value increases, measurements from all four techniques become closer to the LVDT value.

**Figure 13:**
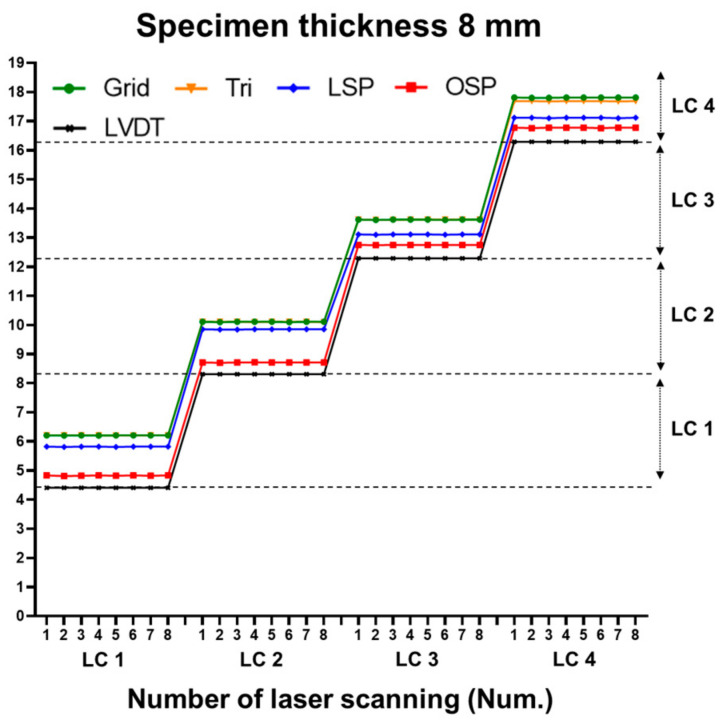
Comparison of vertical displacement with eight repeated laser scans (8 mm specimen).

**Figure 14:**
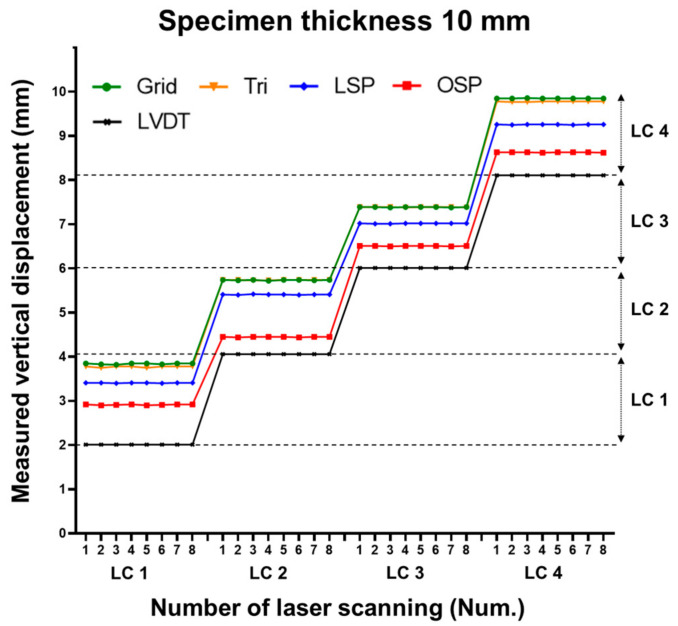
Comparison of vertical displacement with eight repeated laser scans (10 mm specimen).

**Figure 15:**
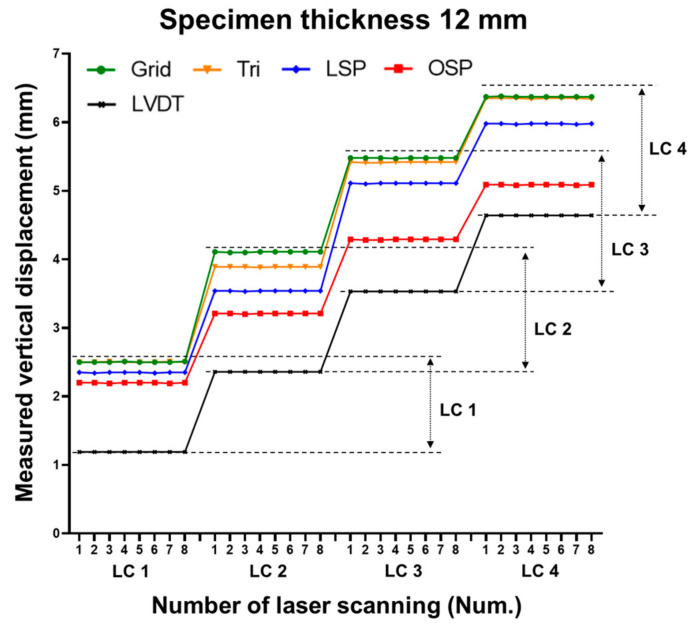
Comparison of vertical displacement with eight repeated laser scans (12 mm specimen).

Three specimens were scanned eight times for each case. Figure 13, Figure 14 and Figure 15 show the vertical displacement values after repeated scanning. After repeated scanning, the measured value was based on the LVDT center point (36 cm), and all four techniques measured the vertical displacement at the 36 cm point. The four techniques showed the same value or between 0.01 and 0.03 mm difference during the eight repeated scans.

The Clock_gettime function was used to check the time for execution of each technique. The Clock_gettime function measures the time in the Linux operating system and can obtain the precise time of the code execution within a specific section. The time was calculated by specifying the range between the beginning and end of the source file for each technique. In Table 10, space relocation is the time required to load data, and displacement estimation is the time required to calculate the displacement value.

As a result of checking the execution time, the TLS (Grid), TLS (Tri), and TLS (LSP) techniques were the time required to load the point cloud. The TLS (OSP) technique took more time, as the point cloud was relocated to the 3D space.

After the data were loaded, the displacement estimation required more than 0.12 s for TLS (Grid), TLS (Tri), and TLS (LSP), but only 0.02 s for TLS (OSP). The three methods (Grid, Tri, and LSP) used all point clouds for displacement calculation, whereas TLS (OSP) converted the points to nodes. Hence, the node displacement estimation method appears to have significantly reduced the time.

**Figure 16:**
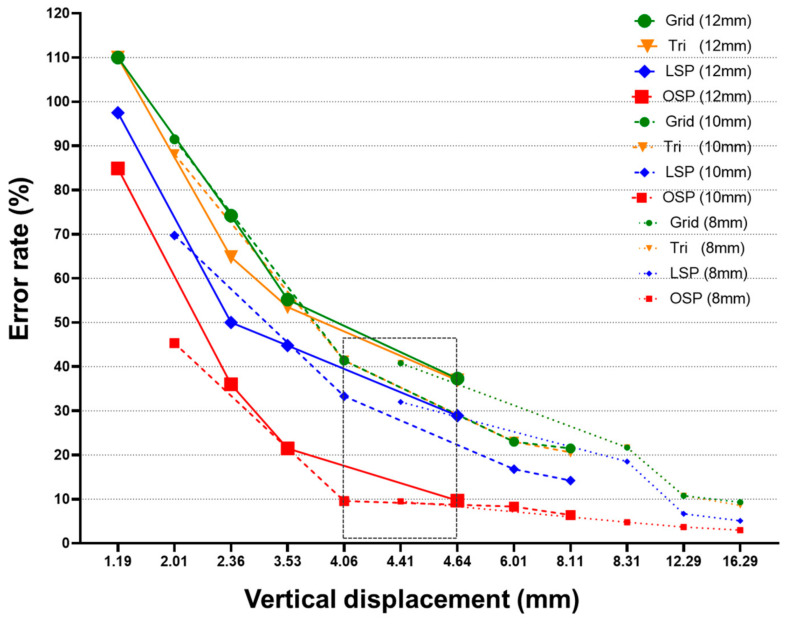
Comparison of error rates for vertical displacement of three specimens based on LVDT.

The error rates were compared based on the LVDT value, which can be seen in Figure 16. The error rates of the all four techniques were found to be less than 10% for displacements greater than or equal to 16 mm. LiDAR equipment has a spot size of 4.5 mm, and the error rate of all four techniques decreased when the displacement value was greater than 4.5 mm. For displacements smaller than 4.5 mm, the error rate gradually increased. The error rate remained within a displacement similar to the spot size for a while, and then it increased rapidly [27,28].

For displacements greater than or equal to 12 mm, only the LSP and OSP techniques had an error rate of less than 10%. For displacements greater than or equal to 4 mm, the OSP technique had an error rate of less than 10%. In Table 11, the section marked in green indicates that the error rate was less than 10% with respect to the LVDT value, and the section marked in yellow indicates the error rate was less than 5%. As a result of the experiment, at a displacement 4.5 mm larger than the spot size of the LiDAR equipment, the OSP technique showed close results to the LVDT value compared to the existing three techniques. In the case of measuring a displacement smaller than the spot size, the error increased significantly, but it was confirmed that the error rate can be measured within 10% up to the 4.06 mm displacement section.

## 4. Discussion

Previous studies obtained analysis results by applying the technique to point cloud data measured through laser scanning. In this study, instead of applying the method directly to the point cloud, the points were rearranged in a three-dimensional space, and nodes were created to calculate the node movement displacement rather than using distance estimation methods between points. The proposed method reprocesses the point cloud data, and the data processing time required is more than the analysis time. Therefore, future research should aim to shorten the data processing time such that the technique can be applied in practice.

## 5. Conclusions

This study proposes a LiDAR-based displacement estimation technique for measuring the total displacement of infrastructure and civil structures. Point cloud data measured through the LiDAR system are arranged in a three-dimensional space and implemented in the form of nodes, where each node is connected via a parent-child relationship. Connected nodes containing location information in 3D space were developed to estimate the vertical displacement of the structure by searching for the location of the node.

The proposed method applied a static load to three specimens (with thicknesses of 8, 10, and 12 mm) and compared the LVDT value and estimated value based on laser scanning. Four laser scanning-based methodologies were employed: Grid, Tri, LSP, and OSP. The displacement estimation results of the four techniques showed that the error rate at displacements greater than or equal to 16 mm was less than 10%. At displacements of 12 mm or more, the LSP and OSP techniques showed an error rate of less than 10%. At displacements of 8 mm or more, the OSP technique showed an error rate of less than 10%. It was confirmed that the OSP technique had the highest accuracy among the four techniques.

The execution time is an important factor because the amount of point cloud data obtained from the structure is large. The execution time of the four techniques was measured by dividing the total time into the time required to load the data and the time required to calculate the displacement. The first three techniques (Grid, Tri, and LSP) required approximately 0.24 s to load and register point cloud data. The OSP technique required approximately 1.5 s, including the time required for reprocessing owing to the conversion of points to nodes. The OSP method required 0.04 s for the displacement estimation after data placement was completed. The displacement estimation times of the first three methods (Grid, Tri, and LSP) were in the range of 0.36 to 0.39 s; thus, displacement estimation using nodes is faster than that using the point cloud.

Therefore, the proposed method proved that the accuracy of displacement estimation can be improved, although it requires time for structural displacement analysis. This study proposed a technique to estimate the displacement of a structure in 3D space. The proposed method can be used for stability evaluation, even for civil engineering structures that are difficult to access.

## Figures and Tables

**Table 1:** Specifications of the terrestrial laser scanner.

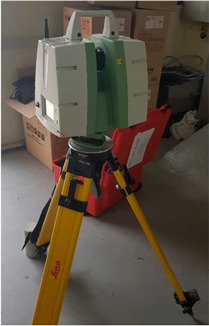	Model		Leica ScanStation C5
	Measurement distance		300 m
	Scan resolution	Spot size	From 0 to 50 m: 4.5 mm (FWHH-based), 7 mm (Gaussian-based)
		Point spacing	Fully selectable horizontal and vertical; <1 mm minimum spacing, through full range; single-point dwell capacity.
	Range accuracy		35 m at 300 m
	Precision		2 mm
	Speed		50,000 point/s
	Range		Horizontal 360° (max), Vertical 270° (max)
	Laser Class		3R (IEC 60825-1)
	Memory		80 GB

**Table 2:** Details of SS400 specimen used in the experiment.

Specimen	SS 400		
	Length (mm)	Width (mm)	Thickness (mm)
1	1000	200	8
2	1000	200	10
3	1400	200	12

**Table 3:** Comparison of theoretical value and actual LVDT value.

Load	Weight	Specimen Thickness	Theoretical Value (mm)	LVDT (mm)	Difference Value (mm)
LC 1	0.8 kN	8 mm	3.56	4.41	−0.85
		10 mm	1.82	2.01	−0.19
		12 mm	1.05	1.19	−0.14
LC 2	1.6 kN	8 mm	7.11	8.31	−1.2
		10 mm	3.64	4.06	−0.42
		12 mm	2.11	2.36	−0.25
LC 3	2.4 kN	8 mm	10.67	12.29	−1.62
		10 mm	5.46	6.01	−0.55
		12 mm	3.16	3.53	−0.37
LC 4	3.2 kN	8 mm	14.22	16.29	−2.07
		10 mm	7.28	8.11	−0.83
		12 mm	4.21	4.64	−0.43

**Table 4:** LVDT measurement results for three specimens.

Thickness	Loading	LVDT Value		
		LVDT 1	LVDT 2	LVDT 3
8 mm specimen	LC 1	3.18	4.41	2.93
	LC 2	5.96	8.31	5.41
	LC 3	8.76	12.29	7.89
	LC 4	11.52	16.29	10.33
10 mm specimen	LC 1	1.43	2.01	1.26
	LC 2	2.91	4.06	2.56
	LC 3	4.31	6.01	3.78
	LC 4	5.81	8.11	5.09
12 mm specimen	LC 1	0.85	1.19	0.74
	LC 2	1.67	2.36	1.48
	LC 3	2.51	3.53	2.22
	LC 4	3.31	4.64	2.92

**Table 5:** Octree spatial division and node displacement algorithm.

**Input** Laser scan binary file (index, value (point, depth, height, width))	**Input** Node displacement check (Ref.octree model, Src.octree model)
**Step 1**	**Step 2**
1: resolution *R* = Space segmentation level 2: **generate** new octree (*R*) 3: **while** (index! = end) 4: **read** Binary data → value 5: **mark** value → empty or full 6: **if** (value == full) 7: octree → Insert Node(value) 8: **else** octree → Delete Node(value) 9: **end if** 10: **next index** 11: **end while** 12: **write** octree → Complete Binary() 13: **end** octree 14: **return** octree storage file	1: **lookup** Ref.model containing Src.model 2: **while** (index! = end) 3: get next closest child (*Ref*, *Src*) 4: Node *C* = Src.index 5: **if** *C* == outside of Node 6: return 0 7: **else** 8: **if** *C* == inside of Node 9: return Distance Calculate (*Ref*, *Src*) 10: **else** 11: find closet node (*Ref*) 12: return Distance Calculate (*Ref*, *Src*) 13: **end if** 14: **end if** 15: **end while** 16: **return** Distance result file

**Table 6:** Performance information of computer for data analysis.

Unit	Specification	Set
CPU	8-Core Intel Xeon E5-2670 (2.6 GHz, 20 MB L3, 8.0GT/sec QPI)	1
RAM	8GB DDR3 ECC-Registered/PC3-12800	8
HDD	SATA 1 TB 32 MB Buffered/7200 rpm	1
POWER	865 W AC power supply w/PFC	1
OS	Ubuntu 12.04.2 LTS (Linux)/64-bit	-

**Table 7:** Octree inner node visualization and node transformation area (10 mm specimen).

Case	Specimen Thickness (10 mm)		
LC 0 (0 kN)	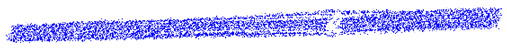		
LC 1 (0.8 kN)	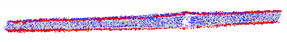	LC 2 (1.6 kN)	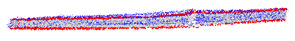
LC 3 (2.4 kN)	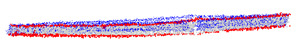	LC 4 (3.2 kN)	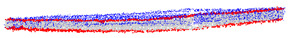

**Table 8:** Octree inner node visualization and node transformation area (8 and 12 mm specimens).

Case	Specimen Thickness (8 mm)	Specimen Thickness (12 mm)
LC 0 (0 kN)	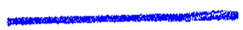	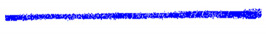
LC 1 (0.8 kN)	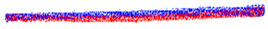	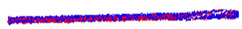
LC 2 (1.6 kN)	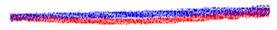	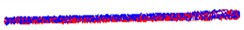
LC 3 (2.4 kN)	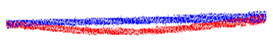	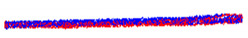
LC 4 (3.2 kN)	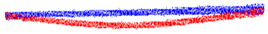	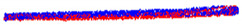

**Table 9:** Estimated value of vertical displacement obtained via four methodologies.

Case	Method	Specimen Thickness		
		8 mm	10 mm	12 mm
LC 1 (0.8 kN)	Grid	6.21	3.85	2.50
	Tri	6.21	3.78	2.50
	LSP	5.82	3.41	2.35
	OSP	4.83	2.92	2.20
LC 2 (1.6 kN)	Grid	10.11	5.74	4.11
	Tri	10.11	5.74	3.89
	LSP	9.85	5.41	3.54
	OSP	8.71	4.45	3.21
LC 3 (2.4 kN)	Grid	13.62	7.39	5.48
	Tri	13.62	7.39	5.42
	LSP	13.11	7.02	5.11
	OSP	12.75	6.51	4.29
LC 4 (3.2 kN)	Grid	17.81	9.85	6.37
	Tri	17.69	9.78	6.35
	LSP	17.12	9.26	5.98
	OSP	16.78	8.63	5.09

**Table 10:** Comparison of execution time between the four methodologies.

Method	Points	Running Time		Total Execution Time (s)
		Space Relocation (s)	Displacement Estimation (s)	
Grid	3527~3673	0.24	0.12	0.36
Tri		0.24	0.15	0.39
LSP		0.24	0.15	0.39
OSP		1.49	0.04	1.53

**Table 11:** Comparison of error rates between LVDT and the four methodologies. Color display of error rate (**Green**: within 10%, **Yellow**: within 5%).

Case	8 mm				10 mm				12 mm			
	LVDT (mm)				LVDT (mm)				LVDT (mm)			
	Grid	Tri	LSP	OSP	Grid	Tri	LSP	OSP	Grid	Tri	LSP	OSP
LC1	−4.41				−2.01				−1.19			
	40.8	40.8	32.0	9.5	91.5	88.1	69.7	45.3	110.1	110.1	97.5	84.9
LC2	−8.31				−4.06				−2.36			
	21.7	21.7	18.5	4.8	41.4	41.4	33.3	9.6	74.2	64.8	50.0	36.0
LC3	−12.29				−6.01				−3.53			
	10.8	10.8	6.7	3.7	23.0	23.0	16.8	8.3	55.2	53.5	44.8	21.5
LC4	−16.29				−8.11				−4.64			
	9.3	8.6	5.1	3.0	21.5	20.6	14.2	6.4	37.3	36.9	28.9	9.7

## References

[1] Riveiro B., DeJong M., Conde B. (2016). Automated processing of large point clouds for structural health monitoring of masonry arch bridges. Autom. Constr..

[2] Xiongyao X., Mingrui A., Jiamin H., Biao Z. (2018). Automatic Processing Method for Deformation Monitoring of Circle Tunnels Based on 3D LiDAR Data. Preprints.

[3] Cabaleiro M., Lindenbergh R., Gard W.F., Arias P. (2017). Algorithm for automatic detection and analysis of cracks in timber beams form LiDAR data. Constr. Build. Mater..

[4] Aloisio A., Pasca D.P., Alaggio R., Fragiacomo M. (2020). Bayesian estimate of the elastic modulus of concrete box girders from dynamic identification: A statistical framework for the A24 motorway in Italy. Struct. Infrastruct. Eng..

[5] Hariri-Ardebili M.A., Sanchez L., Rezakhani R. (2020). Aging of Concrete Structures and Infrastructures: Causes, Consequences, and Cures (C3). Adv. Mater. Sci. Eng..

[6] Beger R., Gedrange C., Hecht R., Neubert M. (2011). Data fusion of extremely high resolution aerial imagery and LiDAR data for automated railroad centre line reconstruction. ISPRS J. Photogramm. Remote Sens..

[7] Maru M.B., Lee D., Cha G., Park S. (2020). Beam Deflection Monitoring Based on a Genetic Algorithm Using Lidar Data. Sensors.

[8] Park H.S., Lee H.M., Adeli H., Lee I. (2007). A New Approach for Health Monitoring of Structures: Terrestrial Laser Scanning. Comput. Civ. Infrastruct. Eng..

[9] Khuc T., Catbas F.N. (2016). Completely contactless structural health monitoring of real-life structures using cameras and computer vision. Struct. Control Health Monit..

[10] Sony S., LaVenture S., Sadhu A. (2019). A literature review of next-generation smart sensing technology in structural health monitoring. Struct. Control Health Monit..

[11] Feng D., Feng M.Q. (2017). Experimental validation of cost-effective vision-based structural health monitoring. Mech. Syst. Signal Process..

[12] Burca G.E., Jerome J.G. (2017). Laser-based surface damage detection and quantification using predicted surface properties. Autom. Constr..

[13] Kang D., Lee H., Park H.S., Lee I. (2007). Computing Method for Estimating Strain and Stress of Steel Beams Using Terrestrial Laser Scanning and FEM. Key Eng. Mater..

[14] Kwiatkowski J., Anigacz W., Beben D. (2020). Comparison of Non-Destructive Techniques for Technological Bridge Deflection Testing. Materials.

[15] Artese S., Zinno R. (2020). TLS for Dynamic Measurement of the Elastic Line of Bridges. Appl. Sci..

[16] Cho S., Park S., Cha G., Oh T.K. (2018). Development of Image Processing for Crack Detection on Concrete Structures through Terrestrial Laser Scanning Associated with the Octree Structure. Appl. Sci..

[17] Wu B., Yu B., Yue W., Shu S., Tan W., Hu C., Huang Y., Wu J., Liu H. (2013). A Voxel-Based Method for Automated Identification and Morphological Parameters Estimation of Individual Street Trees from Mobile Laser Scanning Data. Remote Sens..

[18] Lantsoght E.O., Van Der Veen C., De Boer A., Hordijk D.A. (2017). State-of-the-art on load testing of concrete bridges. Eng. Struct..

[19] Keightley K.E., Bawden G.W. (2010). 3D volumetric modeling of grapevine biomass using Tripod LiDAR. Comput. Electron. Agric..

[20] Nowak R., Orłowicz R., Rutkowski R. (2020). Use of TLS (LiDAR) for Building Diagnostics with the Example of a Historic Building in Karlino. Buildings.

[21] Jo B.W., Lee Y.S., Jo J.H., Khan R.M.A. (2018). Computer Vision-Based Bridge Displacement Measurements Using Rotation-Invariant Image Processing Technique. Sustainability.

[22] Wahbeh A.M., Caffrey J.P., Masri S.F. (2003). A vision-based approach for the direct measurement of displacements in vibrating systems. Smart Mater. Struct..

[23] Notbohm J., Rosakis A., Kumagai S., Xia S., Ravichandran G. (2013). Three-dimensional Displacement and Shape Measurement with a Diffraction-assisted Grid Method. Strain.

[24] Gawronek P., Makuch M., Mitka B., Gargula T. (2019). Measurements of the Vertical Displacements of a Railway Bridge Using TLS Technology in the Context of the Upgrade of the Polish Railway Transport. Sensors.

[25] Gordon S.J., Lichti D.D. (2007). Modeling Terrestrial Laser Scanner Data for Precise Structural Deformation Measurement. J. Surv. Eng..

[26] Hornung A., Wurm K.M., Bennewitz M., Stachniss C., Burgard W. (2013). OctoMap: An efficient probabilistic 3D mapping framework based on octrees. Auton. Robot..

[27] Lõhmus H., Ellmann A., Märdla S., Idnurm S. (2017). Terrestrial laser scanning for the monitoring of bridge load tests—Two case studies. Surv. Rev..

[28] Gawronek P., Makuch M. (2019). TLS Measurement during Static Load Testing of a Railway Bridge. ISPRS Int. J. Geo-Inf..

